# Risk factors associated with failing pre-transmission assessment surveys (pre-TAS) in lymphatic filariasis elimination programs: Results of a multi-country analysis

**DOI:** 10.1371/journal.pntd.0008301

**Published:** 2020-06-01

**Authors:** Clara R. Burgert-Brucker, Kathryn L. Zoerhoff, Maureen Headland, Erica A. Shoemaker, Rachel Stelmach, Mohammad Jahirul Karim, Wilfrid Batcho, Clarisse Bougouma, Roland Bougma, Biholong Benjamin Didier, Nko'Ayissi Georges, Benjamin Marfo, Jean Frantz Lemoine, Helena Ullyartha Pangaribuan, Eksi Wijayanti, Yaya Ibrahim Coulibaly, Salif Seriba Doumbia, Pradip Rimal, Adamou Bacthiri Salissou, Yukaba Bah, Upendo Mwingira, Andreas Nshala, Edridah Muheki, Joseph Shott, Violetta Yevstigneyeva, Egide Ndayishimye, Margaret Baker, John Kraemer, Molly Brady

**Affiliations:** 1 Global Health Division, RTI International, Washington, DC, United States of America; 2 Global Health, Population, and Nutrition, FHI 360, Washington, DC, United States of America; 3 Department of Disease Control, Ministry of Health and Family Welfare, Dhaka, Bangladesh; 4 National Control Program of Communicable Diseases, Ministry of Health, Cotonou, Benin; 5 Lymphatic Filariasis Elimination Program, Ministère de la Santé, Ouagadougou, Burkina Faso; 6 National Onchocerciasis and Lymphatic Filariasis Control Program, Ministry of Health, Yaounde, Cameroon; 7 Neglected Tropical Diseases Programme, Ghana Health Service, Accra, Ghana; 8 Ministry of Health, Port-au-Prince, Haiti; 9 National Institute Health Research & Development, Ministry of Health, Jakarta, Indonesia; 10 Filariasis Unit, International Center of Excellence in Research, Faculty of Medicine and Odontostomatology, Bamako, Mali; 11 Epidemiology and Disease Control Division, Department of Health Service, Kathmandu, Nepal; 12 Programme Onchocercose et Filariose Lymphatique, Ministère de la Santé, Niamey, Niger; 13 National Neglected Tropical Disease Program, Ministry of Health and Sanitation, Freetown, Sierra Leone; 14 Neglected Tropical Disease Control Programme, National Institute for Medical Research, Dar es Salaam, Tanzania; 15 IMA World Health/Tanzania NTD Control Programme, Uppsala University, & TIBA Fellow, Dar es Salaam, Tanzania; 16 Programme to Eliminate Lymphatic Filariasis, Ministry of Health, Kampala, Uganda; 17 Division of Neglected Tropical Diseases, Office of Infectious Diseases, Bureau for Global Health, USAID, Washington, DC, United States of America; 18 Georgetown University, Washington, DC, United States of America; Washington University School of Medicine, UNITED STATES

## Abstract

Achieving elimination of lymphatic filariasis (LF) as a public health problem requires a minimum of five effective rounds of mass drug administration (MDA) and demonstrating low prevalence in subsequent assessments. The first assessments recommended by the World Health Organization (WHO) are sentinel and spot-check sites—referred to as pre-transmission assessment surveys (pre-TAS)—in each implementation unit after MDA. If pre-TAS shows that prevalence in each site has been lowered to less than 1% microfilaremia or less than 2% antigenemia, the implementation unit conducts a TAS to determine whether MDA can be stopped. Failure to pass pre-TAS means that further rounds of MDA are required. This study aims to understand factors influencing pre-TAS results using existing programmatic data from 554 implementation units, of which 74 (13%) failed, in 13 countries. Secondary data analysis was completed using existing data from Bangladesh, Benin, Burkina Faso, Cameroon, Ghana, Haiti, Indonesia, Mali, Nepal, Niger, Sierra Leone, Tanzania, and Uganda. Additional covariate data were obtained from spatial raster data sets. Bivariate analysis and multilinear regression were performed to establish potential relationships between variables and the pre-TAS result. Higher baseline prevalence and lower elevation were significant in the regression model. Variables statistically significantly associated with failure (p-value ≤0.05) in the bivariate analyses included baseline prevalence at or above 5% or 10%, use of Filariasis Test Strips (FTS), primary vector of *Culex*, treatment with diethylcarbamazine-albendazole, higher elevation, higher population density, higher enhanced vegetation index (EVI), higher annual rainfall, and 6 or more rounds of MDA. This paper reports for the first time factors associated with pre-TAS results from a multi-country analysis. This information can help countries more effectively forecast program activities, such as the potential need for more rounds of MDA, and prioritize resources to ensure adequate coverage of all persons in areas at highest risk of failing pre-TAS.

## Author summary

Achieving elimination of lymphatic filariasis (LF) as a public health problem requires a minimum of five rounds of mass drug administration (MDA) and being able to demonstrate low prevalence in several subsequent assessments. LF elimination programs implement sentinel and spot-check site assessments, called pre-TAS, to determine whether districts are eligible to implement more rigorous population-based surveys to determine whether MDA can be stopped or if further rounds are required. Reasons for failing pre-TAS are not well understood and have not previously been examined with data compiled from multiple countries. For this analysis, we analyzed data from routine USAID and WHO reports from Bangladesh, Benin, Burkina Faso, Cameroon, Ghana, Haiti, Indonesia, Mali, Nepal, Niger, Sierra Leone, Tanzania, and Uganda. In a model that included multiple variables, high baseline prevalence and lower elevation were significant. In models comparing only one variable to the outcome, the following were statistically significantly associated with failure: higher baseline prevalence at or above 5% or 10%, use of the FTS, primary vector of *Culex*, treatment with diethylcarbamazine-albendazole, lower elevation, higher population density, higher Enhanced Vegetation Index, higher annual rainfall, and six or more rounds of mass drug administration. These results can help national programs plan MDA more effectively, e.g., by focusing resources on areas with higher baseline prevalence and/or lower elevation.

## Introduction

Lymphatic filariasis (LF), a disease caused by parasitic worms transmitted to humans by mosquito bite, manifests in disabling and stigmatizing chronic conditions including lymphedema and hydrocele. To eliminate LF as a public health problem, the World Health Organization (WHO) recommends two strategies: reducing transmission through annual mass drug administration (MDA) and reducing suffering through ensuring the availability of morbidity management and disability prevention services to all patients [1]. For the first strategy, eliminating LF as a public health problem is defined as a ‘reduction in measurable prevalence in infection in endemic areas below a target threshold at which further transmission is considered unlikely even in the absence of MDA’ [2]. As of 2018, 14 countries have eliminated LF as a public health problem while 58 countries remain endemic for LF [3].

The road to elimination as a public health problem has several milestones. First, where LF prevalence at baseline has exceeded 1% as measured either through microfilaremia (Mf) or antigenemia (Ag), MDA is implemented and treatment coverage is measured in all implementation units, which usually correspond to districts. Implementation units must complete at least five rounds of ‘effective’ treatment, i.e. treatment with a minimum coverage of 65% of the total population. Then, WHO recommends sentinel and spot-check site assessments—referred to as pre-transmission assessment surveys (pre-TAS)—in each implementation unit to determine whether prevalence in each site is less than 1% Mf or less than 2% Ag [4]. Next, if these thresholds are met, national programs can progress to the first transmission assessment survey (TAS). The TAS is a population-based cluster or systematic survey of six- and seven-year-old children to assess whether transmission has fallen below the threshold at which infection is believed to persist. TAS is conducted at least three times, with two years between each survey. TAS 1 results determine if it is appropriate to stop MDA or whether further rounds are required. Finally, when TAS 2 and 3 also fall below the set threshold in every endemic implementation unit nationwide and morbidity criteria have been fulfilled, the national program submits a dossier to WHO requesting that elimination be officially validated.

Pre-TAS include at least one sentinel and one spot-check site per one million population. Sentinel sites are established at the start of the program in villages where LF prevalence was believed to be relatively high. Spot-check sites are villages not previously tested but purposively selected as potentially high-risk areas due to original high prevalence, low coverage during MDA, high vector density, or other factors [4]. At least six months after MDA implementation, data are collected from a convenience sample of at least 300 people over five years old in each site. Originally, Mf was recommended as the indicator of choice for pre-TAS, assessed by blood smears taken at the time of peak parasite periodicity [4]. WHO later recommended the use of circulating filarial antigen rapid diagnostic tests, BinaxNow immunochromatographic card tests (ICTs), and after 2016, Alere Filariasis Test Strips (FTS), because they are more sensitive, easier to implement, and more flexible about time of day that blood can be taken [5].

When a country fails to meet the established thresholds in a pre-TAS, they must implement at least two more rounds of MDA. National programs need to forecast areas that might fail pre-TAS and need repeated MDA, so that they can inform the community and district decision-makers of the implications of pre-TAS failure, including the need for continued MDA to lower prevalence effectively. In addition, financial and human resources must be made available for ordering drugs, distributing drugs, supervision and monitoring to implement the further MDA rounds. Ordering drugs and providing MDA budgets often need to be completed before the pre-TAS are implemented, so contingency planning and funding are important to ensure rounds of MDA are not missed.

This study aims to understand which factors are associated with the need for additional rounds of MDA as identified by pre-TAS results using programmatic data from 13 countries. The factors associated with failing pre-TAS are not well understood and have not previously been examined at a multi-country scale in the literature. We examine the association between pre-TAS failure and baseline prevalence, parasites, environmental factors, MDA implementation, and pre-TAS implementation. Understanding determinants of pre-TAS failure will help countries identify where elimination may be most difficult and prioritize the use of limited LF elimination resources.

## Methods

This is a secondary data analysis using existing data, collected for programmatic purposes. Data for this analysis come from 568 districts in 13 countries whose LF elimination programs were supported by the United States Agency for International Development (USAID) through the ENVISION project, led by RTI International, and the END in Africa and END in Asia projects, led by FHI 360. These countries are Bangladesh, Benin, Burkina Faso, Cameroon, Ghana, Haiti, Indonesia, Mali, Nepal, Niger, Sierra Leone, Tanzania, and Uganda. The data represent all pre-TAS funded by USAID from 2012 to 2017 and, in some cases, surveys funded by host government or other non-United States government funders. Because pre-TAS data were collected as part of routine program activities in most countries, in general, ethical clearance was not sought for these surveys. Our secondary analysis only included the aggregated survey results and therefore did not constitute human subjects research; no ethical approval was required.

Building on previous work, we delineated five domains of variables that could influence pre-TAS outcomes: prevalence, agent, environment, MDA, and pre-TAS implementation (Table 1) [6–8]. We prioritized key concepts that could be measured through our data or captured through publicly available global geospatial data sets.

**Table 1:** Categorization of potential factors influencing pre-TAS results.

Domain	Factor	Covariate	Description	Reference Group	Summary statistic	Temporal Resolution	Source
Prevalence	Baseline prevalence	5% cut off	Maximum reported mapping or baseline sentinel site prevalence	<5%	Maximum	Varies	Programmatic data
Prevalence	Baseline prevalence	10% cut off	Maximum reported mapping or baseline sentinel site prevalence	<10%	Maximum	Varies	Programmatic data
Agent	Parasite	Parasite	Predominate parasite in district	*W*. *bancrofti* & mixed	Binary value	2018	Programmatic data
Environment	Vector	Vector	Predominate vector in district	*Anopheles* & *Mansonia*	Binary value	2018	Country expert
Environment	Geography	Elevation	Elevation measured in meters	>350	Mean	2000	CGIAR-CSI SRTM [9]
Environment	Geography	District area	Area measured in km^2^	>2,500	Maximum sum	Static	Programmatic data
Environment	Climate	EVI	Enhanced vegetation index	> 0.3	Mean	2015	MODIS [10]
Environment	Climate	Rainfall	Annual rainfall measured in mm	≤ 700	Mean	2015	CHIRPS [11]
Environment	Socio-economic	Population density	Number of people per km^2^	≤ 100	Mean	2015	WorldPop [12]
Environment	Socio-economic	Nighttime lights	Nighttime light index from 0 to 63	>1.5	Mean	2015	VIIRS [13]
Environment	Co-endemicity	Co-endemic for onchocerciasis	Part or all of district is also endemic for onchocerciases	Non-endemic	Binary value	2018	Programmatic data
MDA	Drug efficacy	Drug package	DEC-ALB or IVM-ALB	DEC-ALB	Binary value	2018	Programmatic data
MDA	Implementation of MDA	Coverage	Median MDA coverage for last 5 rounds	≥ 65%	Median	Varies	Programmatic data
MDA	Implementation of MDA	Sufficient rounds	Number of rounds of sufficient (≥ 65% coverage) in last 5 years	≥ 3	Count	Varies	Programmatic data
MDA	Implementation of MDA	Number of rounds	Maximum number of recorded rounds of MDA	≥ 6	Maximum	Varies	Programmatic data
Pre-TAS implementation	Quality of survey	Diagnostic method	Using Mf or Ag	Mf	Binary value	Varies	Programmatic data
Pre-TAS implementation	Quality of survey	Diagnostic test	Using Mf, ICT, or FTS	Mf	Categorical	Varies	Programmatic data

EVI = enhanced vegetation index; MDA = mass drug administration; TAS = transmission assessment survey; DEC = diethylcarbamazine; ALB = albendazole; IVM = ivermectin; Mf = microfilaremia; Ag = antigenemia; FTS = Filariasis Test Strips; ICT = Immunochromatographic Card Test

### Data sources

Information on baseline prevalence, MDA coverage, the number of MDA rounds, and pre-TAS information (month and year of survey, district, site name, and outcome) was gathered through regular reporting for the USAID-funded NTD programs (ENVISION, END in Africa, and END in Asia). These data were augmented by other reporting data such as the country’s dossier data annexes, the WHO Preventive Chemotherapy and Transmission Control Databank, and WHO reporting forms. Data were then reviewed by country experts, including the Ministry of Health program staff and implementing program staff, and updated as necessary. Data on vectors were also obtained from country experts. The district geographic boundaries were matched to geospatial shapefiles from the ENVISION project geospatial data repository, while other geospatial data were obtained through publicly available sources (Table 1).

### Outcome and covariate variables

The outcome of interest for this analysis was whether a district passed or failed the pre-TAS. Failure was defined as any district that had at least one sentinel or spot-check site with a prevalence higher than or equal to 1% Mf or 2% Ag [4].

Potential covariates were derived from the available data for each factor in the domain groups listed in Table 1. New dichotomous variables were created for all variables that had multiple categories or were continuous for ease of interpretation in models and use in program decision-making. Cut-points for continuous variables were derived from either *a priori* knowledge or through exploratory analysis considering the mean or median value of the dataset, looking to create two groups of similar size with logical cut-points (e.g. rounding numbers to whole numbers). All the variables derived from publicly available global spatial raster datasets were summarized to the district level in ArcGIS Pro using the “zonal statistics” tool. The final output used the continuous value measuring the mean pixel value for the district for all variables except geographic area. Categories for each variable were determined by selecting the mean or median dataset value or cut-off used in other relevant literature [7]. The following section describes the variables that were included in the final analysis and the final categorizations used.

#### Baseline prevalence

Baseline prevalence can be assumed as a proxy for local transmission conditions [14] and correlates with prevalence after MDA [14–20]. Baseline prevalence for each district was measured by either blood smears to measure Mf or rapid diagnostic tests to measure Ag. Other studies have modeled Mf and Ag prevalence separately, due to lack of a standardized correlation between the two, especially at pre-MDA levels [21,22]. However, because WHO mapping guidance states that MDA is required if either Mf or Ag is ≥1% and there were not enough data to model each separately, we combined baseline prevalence values regardless of diagnostic test used. We created two variables for use in the analysis (1) using the cut-off of <5% or ≥5% (dataset median value of 5%) and (2) using the cut-off of <10% or ≥10%.

#### Agent

In terms of differences in transmission dynamics by agent, research has shown that *Brugia* spp. are more susceptible to the anti-filarial drug regimens than *Wuchereria bancrofti* parasites [23]. Thus, we combined districts reporting *B*. *malayi* and *B*. *timori* and compared them to areas with *W*. *bancrofti* or mixed parasites. Two variables from other domains were identified in exploratory analyses to be highly colinear with the parasite, and thus we considered them in the same group of variables for the final regression models. These were variables delineating vectors (*Anopheles* or *Mansonia* compared to *Culex*) from the environmental domain and drug package [ivermectin-albendazole (IVM-ALB) compared to diethylcarbamazine-albendazole (DEC-ALB)] from the MDA domain.

#### Environment

LF transmission intensity is influenced by differing vector transmission dynamics, including vector biting rates and competence, and the number of individuals with microfilaria [21,24,25]. Since vector data are not always available, previous studies have explored whether environmental variables associated with vector density, such as elevation, rainfall, and temperature, can be used to predict LF prevalence [8,21,26–31]. We included the district area and elevation in meters as geographic variables potentially associated with transmission intensity. In addition, within the climate factor, we included Enhanced Vegetation Index (EVI) and rainfall variables. EVI measures vegetation levels, or “greenness,” where a higher index value indicates a higher level of “greenness.”

We included the socio-economic variable of population density, as it has been positively associated with LF prevalence in some studies [8,27,29], but no significant association has been found in others [30]. Population density could be correlated with vector, as in eastern African countries LF is mostly transmitted by *Culex* in urban areas and by *Anopheles* in rural areas [32]. Additionally, inclusion of the satellite imagery of nighttime lights data is another a proxy for socio-economic status [33].

Finally, all or parts of districts that are co-endemic with onchocerciasis may have received multiple rounds of MDA with ivermectin before LF MDA started, which may have lowered LF prevalence in an area [34–36]. Thus, we included a categorical variable to distinguish if districts were co-endemic with onchocerciasis.

#### MDA

Treatment effectiveness depends upon both drug efficacy (ability to kill adult worms, ability to kill Mf, drug resistance, drug quality) and implementation of MDA (coverage, compliance, number of rounds) [14,16]. Ivermectin is less effective against adult worms than DEC, and therefore it is likely that Ag reduction is slower in areas using ivermectin instead of DEC in MDA [37]. Models also have shown that MDA coverage affects prevalence, although coverage has been defined in various ways, such as median coverage, number of rounds, or individual compliance [14–16,20,38–40]. Furthermore, systematic non-compliance, or population sub-groups which consistently refuse to take medicines, has been shown to represent a threat to elimination [41,42].

We considered three approaches when analyzing the MDA data: median MDA coverage in the most recent 5 rounds, number of rounds with sufficient coverage in the most recent 5 rounds, and count of the total number of rounds. MDA coverage is considered sufficient at or above 65% of the total population who were reported to have ingested the drugs; this was used as the cut point for MDA median coverage for the most recent 5 rounds. The ‘rounds of sufficient coverage’ variable was categorized as having 2 or fewer rounds compared to 3 or more sufficient rounds. The ‘total number of MDA rounds’ variable was categorized at 5 or fewer rounds compared to 6 or more rounds ever documented in that district.

#### Pre-TAS implementation

Pre-TAS results can be influenced by the implementation of the survey itself, including the use of a particular diagnostic test, the selection of sites, the timing of survey, and the appropriate application of methods for population recruitment and diagnostic test adminstration. We included two variables in the pre-TAS implementation domain: `type of diagnostic method used’ and `diagnostic test used.’ The ‘type of diagnostic method used’ variable categorized districts by either using Mf or Ag. The ‘diagnostic test used’ variable examined Mf (reference category) compared to ICT and compared to FTS (categorical variable with 3 values). This approach was used to compare each test to each other. Countries switched from ICT to FTS during 2016, while Mf testing continued to be used throughout the time period of study.

### Data inclusion criteria

The dataset, summarized at the district level, included information from 568 districts where a pre-TAS was being implemented for the first time. A total of 14 districts were removed from the final analysis due to missing data related to the following points: geospatial boundaries (4), baseline prevalence (4), and MDA coverage (6). The final analysis dataset had 554 districts.

### Statistical analysis and modeling

Statistical analysis and modeling were done with Stata MP 15.1 (College Station, TX). Descriptive statistics comparing various variables to the principle outcome were performed. Significant differences were identified using a chi-square test. A generalized linear model (GLM) with a log link and binomial error distribution—which estimates relative risks—was developed using forward stepwise modeling methods (called log-binomial model). Models with higher pseudo-r-squared and lower Akaike information criterion (AIC) were retained at each step. Pseudo-r-squared is a value between 0 and 1 with the higher the value, the better the model is at predicting the outcome of interest. AIC values are used to compare the relative quality of models compared to each other; in general, a lower value indicates a better model. Variables were tested by factor group. Once a variable was selected from the group, no other variable in that same group was eligible to be included in the final model due to issues of collinearity and small sample sizes. Interaction between terms in the model was tested after model selection, and interaction terms that modified the original terms’ significance were included in the final model. Overall, the number of potential variables able to be included in the model remained low due to the relatively small number of failure results (13%) in the dataset. Furthermore, the models with more than 3 variables and one interaction term either were unstable (indicated by very large confidence interval widths) or did not improve the model by being significant predictors or by modifying other parameters already in the model. These models were at heightened risk of non-convergence; we limited the number of variables accordingly.

Sensitivity analysis was performed for the final log-binomial model to test for the validity of results under different parameters by excluding some sub-sets of districts from the dataset and rerunning the model. This analysis was done to understand the robustness of the model when (1) excluding all districts in Cameroon, (2) including only districts in Africa, (3) including only districts with *W*. *bancrofti* parasite, and (4) including only districts with *Anopheles* as the primary vector. The sensitivity analysis excluding Cameroon was done for two reasons. First, Cameroon had the most pre-TAS results included, but no failures. Second, 70% of the Cameroon districts included in the analysis are co-endemic for loiasis. Given that diagnostic tests used in LF mapping have since been shown to cross-react with loiasis, there is some concern that these districts might not have been truly LF-endemic [43,44].

## Results

**Fig 1:**
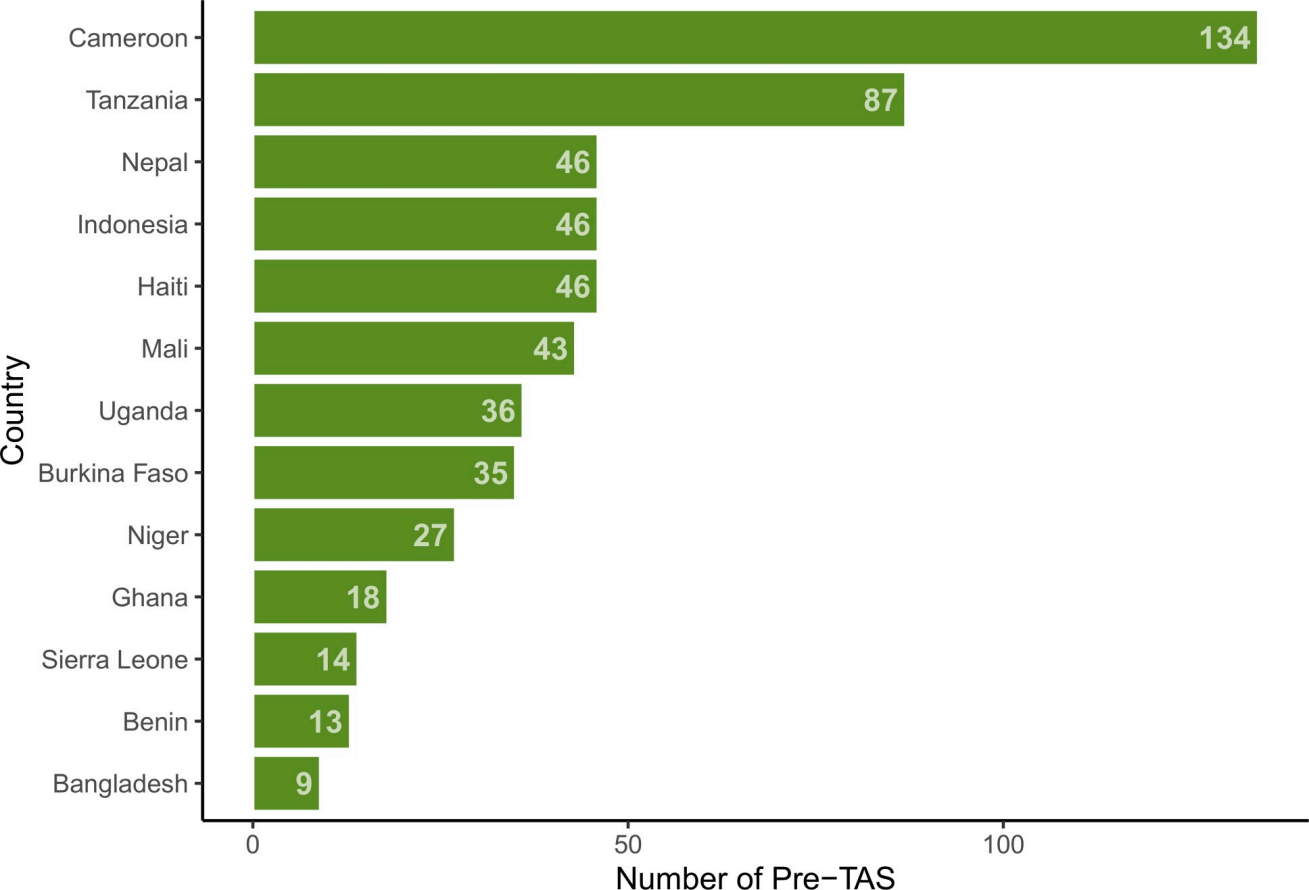
Number of pre-TAS by country.

**Fig 2:**
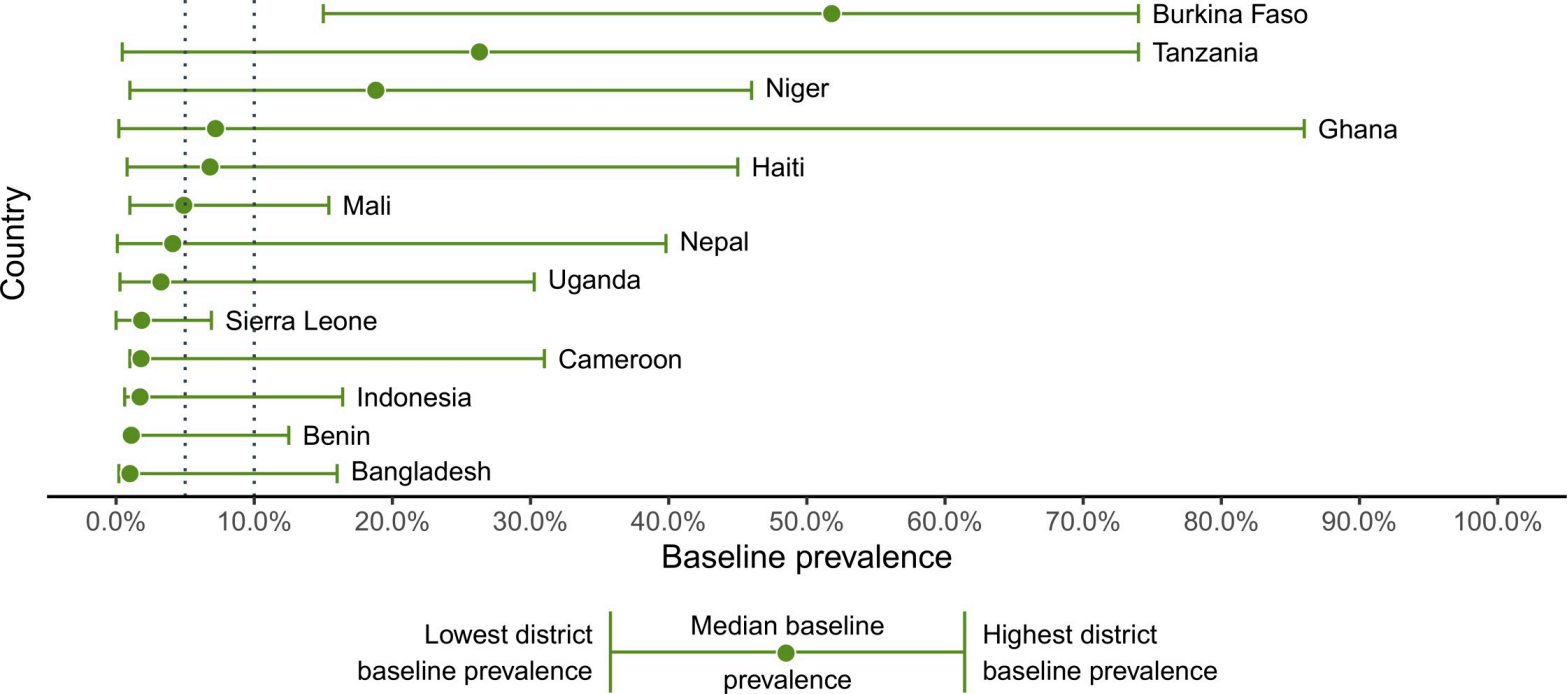
District-level baseline prevalence by country.

The overall pre-TAS pass rate for the districts included in this analysis was 87% (74 failures in 554 districts). Nearly 40% of the 554 districts were from Cameroon (134) and Tanzania (87) (Fig 1). No districts in Bangladesh, Cameroon, Mali, or Uganda failed a pre-TAS in this data set; over 25% of districts in Burkina Faso, Ghana, Haiti, Nepal, and Sierra Leone failed pre-TAS in this data set. Baseline prevalence varied widely within and between the 13 countries. Fig 2 shows the highest, lowest, and median baseline prevalence in the study districts by country. Burkina Faso had the highest median baseline prevalence at 52% and Burkina Faso, Tanzania, and Ghana all had at least one district with a very high baseline of over 70%. In Mali, Indonesia, Benin, and Bangladesh, all districts had baseline prevalences below 20%.

**Fig 3:**
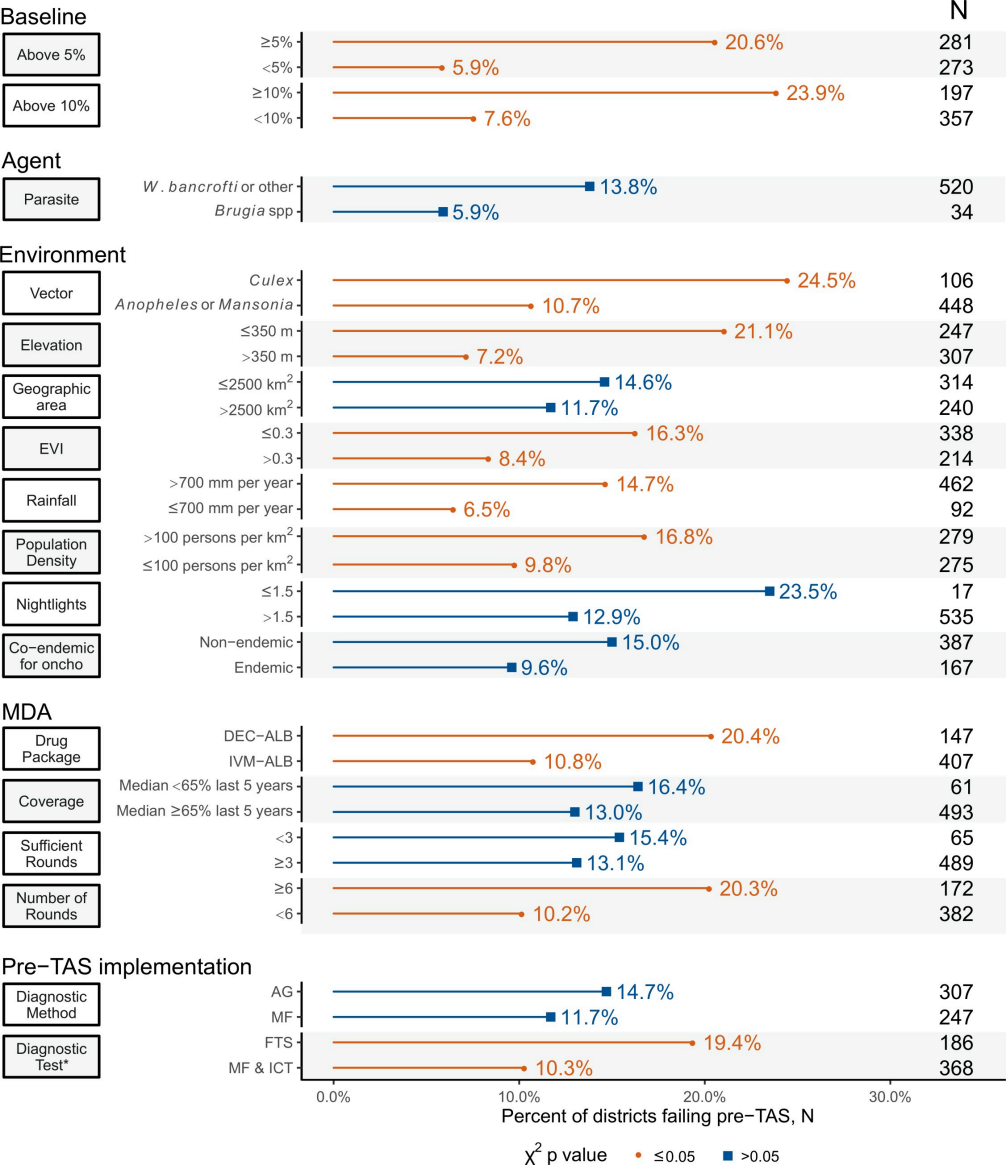
Percent pre-TAS failure by each characteristic (unadjusted).

Fig 3 shows the unadjusted analysis for key variables by pre-TAS result. Variables statistically significantly associated with failure (p-value ≤0.05) included higher baseline prevalence at or above 5% or 10%, FTS diagnostic test, primary vector of *Culex*, treatment with DEC-ALB, higher elevation, higher population density, higher EVI, higher annual rainfall, and six or more rounds of MDA. Variables that were not significantly associated with pre-TAS failure included diagnostic method used (Ag or Mf), parasite, co-endemicity for onchocerciasis, median MDA coverage, and sufficient rounds of MDA.

The final log-binomial model included the variables of baseline prevalence ≥10%, the diagnostic test used (FTS and ICT), and elevation. The final model also included a significant interaction term between high baseline and diagnostic test used.

**Fig 4:**
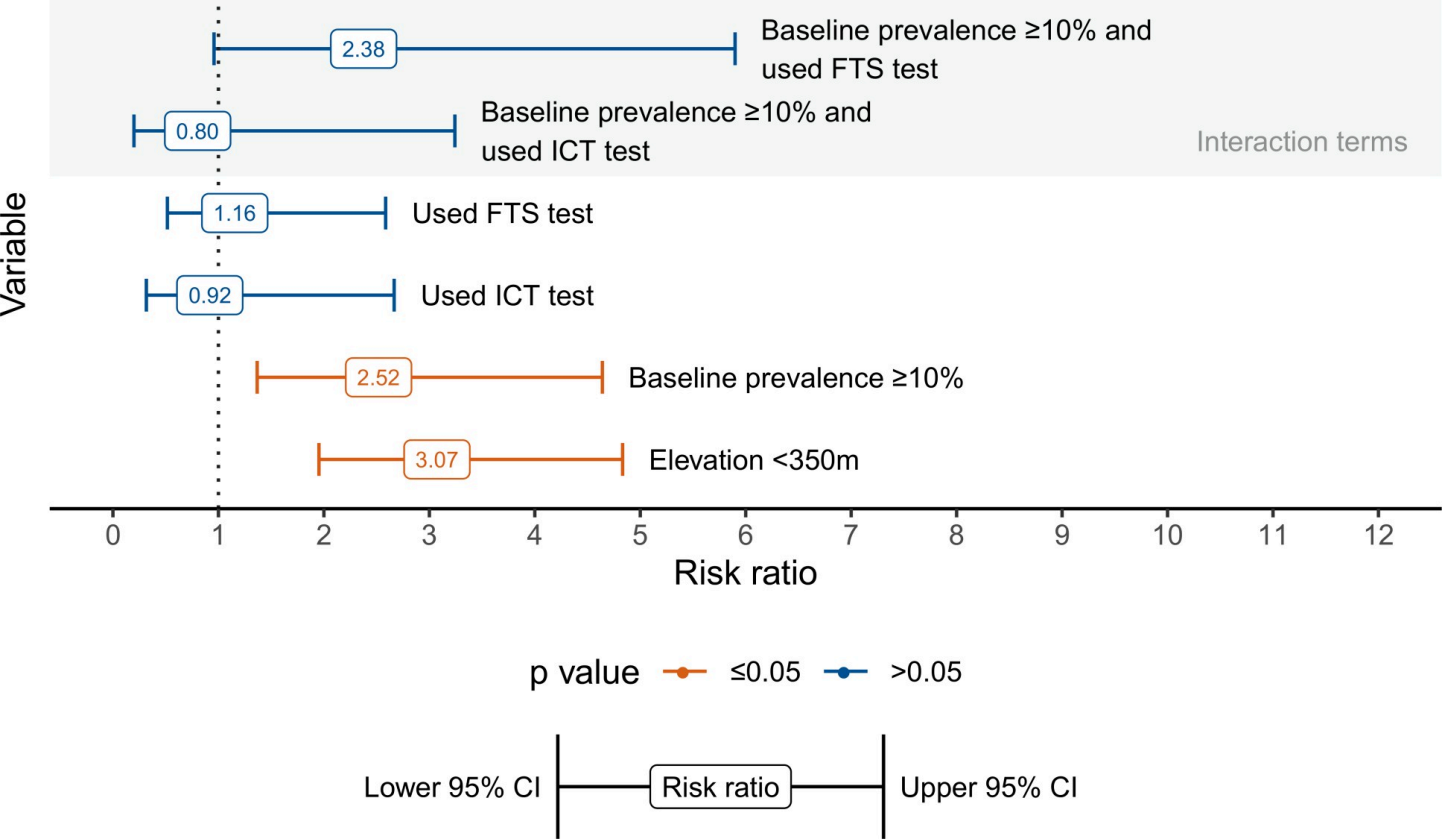
Adjusted risk ratios for pre-TAS failure with 95% Confidence Interval from log-binomial model.

Fig 4 shows the risk ratio results with their corresponding confidence intervals. In a model with interaction between baseline and diagnostic test the baseline parameter was significant while diagnostic test and the interaction term were not. Districts with high baseline had a statistically significant (p-value ≤0.05) 2.52 times higher risk of failure (95% CI 1.37–4.64) compared to those with low baseline prevalence. The FTS diagnostic test or ICT diagnostic test alone were not significant nor was the interaction term. Additionally, districts with an elevation below 350 meters had a statistically significant (p-value ≤0.05) 3.07 times higher risk of failing pre-TAS (95% CI 1.95–4.83).

Sensitivity analyses were conducted using the same model with different subsets of the dataset including (1) all districts except for districts in Cameroon (134 total with no failures), (2) only districts in Africa, (3) only districts with *W*. *bancrofti*, and (4) only districts with *Anopheles* as primary vector. The results of the sensitivity models (Table 2) indicate an overall robust model. High baseline and lower elevation remained significant across all the models. The ICT diagnostic test used remains insignificant across all models. The FTS diagnostic test was positively significant in model 1 and negatively significant in model 4. The interaction term of baseline prevalence and FTS diagnostic test was significant in three models though the estimate was unstable in the *W*. *bancrofti*-only and *Anopheles*-only models (models 3 and 4 respectively), as signified by large confidence intervals.

**Table 2:** Adjusted risk ratios for pre-TAS failure from log-binomial model sensitivity analysis.

		(1)	(2)	(3)	(4)
	Full Model	Without Cameroon districts	Only districts in Africa	Only *W*. *bancrofti* parasite districts	Only Anopheles vector districts
Number of Failures	74	74	44	72	46
Number of total districts	(N = 554)	(N = 420)	(N = 407)	(N = 518)	(N = 414)
Covariate	RR (95% CI)	RR (95% CI)	RR (95% CI)	RR (95% CI)	RR (95% CI)
Baseline prevalence > = 10% & used FTS test	2.38 (0.96–5.90)	1.23 (0.52–2.92)	**14.52 (1.79–117.82)**	**2.61 (1.03–6.61)**	**15.80 (1.95–127.67)**
Baseline prevalence > = 10% & used ICT test	0.80 (0.20–3.24)	0.42 (0.11–1.68)	1.00 (0.00–0.00)	0.88 (0.21–3.60)	1.00 (0.00–0.00)
*+Used FTS test*	*1*.*16 (0*.*52–2*.*59)*	**2.40 (1.12–5.11)**	0.15 (0.02–1.11)	1.03 (0.45–2.36)	0.13 (0.02–0.96)
*+Used ICT test*	*0*.*92 (0*.*32–2*.*67)*	1.47 (0.51–4.21)	0.33 (0.04–2.54)	0.82 (0.28–2.43)	0.27 (0.03–2.04)
*+Baseline prevalence > = 10%*	***2*.*52 (1*.*37–4*.*64)***	**2.42 (1.31–4.47)**	**2.03 (1.06–3.90)**	**2.30 (1.21–4.36)**	**2.01 (1.07–3.77)**
Elevation < 350m	**3.07 (1.95–4.83)**	**2.21 (1.42–3.43)**	**4.68 (2.22–9.87)**	**3.04 (1.93–4.79)**	**3.76 (1.92–7.37)**

NOTE: Table shows adjusted Risk Ratio (RR) of failing pre-TAS.

Gray shading & + denote interaction terms, Bold text denotes statistically significant results with p-values ≤0.05

**Fig 5:**
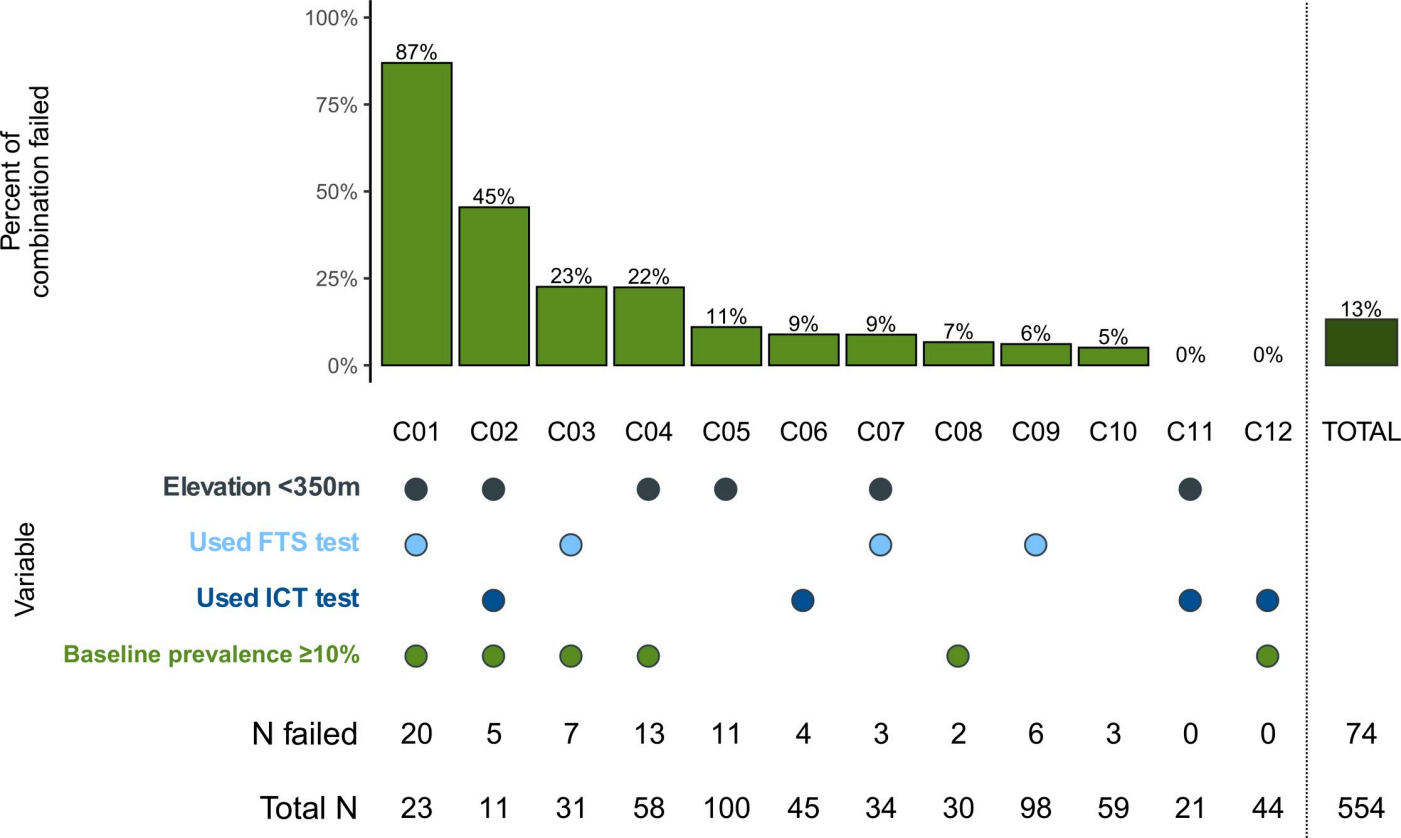
Analysis of failures by model combinations.

Overall 74 districts in the dataset failed pre-TAS. Fig 5 summarizes the likelihood of failure by variable combinations identified in the log-binomial model. For those districts with a baseline prevalence ≥10% that used a FTS diagnostic test and have an average elevation below 350 meters (Combination C01), 87% of the 23 districts failed. Of districts with high baseline that used an ICT diagnostic test and have a low average elevation (C02) 45% failed. Overall, combinations with high baseline and low elevation C01, C02, and C04 accounted for 51% of all the failures (38 of 74).

## Discussion

This paper reports for the first time factors associated with pre-TAS results from a multi-country analysis. Variables significantly associated with failure were higher baseline prevalence and lower elevation. Districts with a baseline prevalence of 10% or more were at 2.52 times higher risk to fail pre-TAS in the final log-binomial model. In the bivariate analysis, baseline prevalence above 5% was also significantly more likely to fail compared to lower baselines, which indicates that the threshold for higher baseline prevalence may be as little as 5%, similar to what was found in Goldberg *et al*., which explored ecological and socioeconomic factors associated with TAS failure [7].

Though diagnostic test used was selected for the final log-binomial model, neither category (FTS or ICT) were significant after interaction with high baseline. FTS alone is significant in the bivariate analysis compared to ICT or Mf. This result is not surprising given previous research which found that FTS was more sensitive than ICT [45].

Elevation was the only environmental domain variable selected for the final log-binomial model during the model selection process, with areas of lower elevation (<350m) found to be at 3.07 times higher risk to fail pre-TAS compared to districts with a higher elevation. Similar results related to elevation were found in previous studies [8,31], including Goldberg *et al*. [7], who used a cutoff of 200 meters. Elevation likely also encompasses some related environmental concepts, such as vector habitat, greenness (EVI), or rainfall, which impact vector chances of survival.

The small number of failures overall prevented the inclusion of a large number of variables in the final log-binomial model. However, other variables that are associated with failure as identified in the bivariate analyses, such as *Culex* vector, higher population density, higher EVI, higher rainfall and more rounds of MDA, should not be discounted when making programmatic decisions. Other models have shown that *Culex* as the predominant vector in a district, compared to *Anopheles*, results in more intense interventions needed to reach elimination [24,41]. Higher population density, which was also found to predict TAS failure [7], could be related to different vector species’ transmission dynamics in urban areas, as well as the fact that MDAs are harder to conduct and to accurately measure in urban areas [46,47]. Both higher enhanced vegetation index (>0.3) and higher rainfall (>700 mm per year) contribute to expansion of vector habitats and population. Additionally, having more than five rounds of MDA before pre-TAS was also statistically significantly associated with higher failure in the bivariate analysis. It is unclear why higher number of rounds is associated with first pre-TAS failure given that other research has shown the opposite [15,16].

All other variables included in this analysis were not significantly associated with pre-TAS failure in our analysis. Goldberg *et al*. found *Brugia* spp. to be significantly associated with failure, but our results did not. This is likely due in part to the small number of districts with *Brugia* spp. in our dataset (6%) compared to 46% in the Goldberg *et al*. article [7]. MDA coverage levels were not significantly associated with pre-TAS failure, likely due to the lack of variance in the coverage data since WHO guidance dictates a minimum of five rounds of MDA with ≥65% epidemiological coverage to be eligible to implement pre-TAS. It should not be interpreted as evidence that high MDA coverage levels are not necessary to lower prevalence.

Limitations to this study include data sources, excluded data, unreported data, misassigned data, and aggregation of results at the district level. The main data sources for this analysis were programmatic data, which may be less accurate than data collected specifically for research purposes. This is particularly true of the MDA coverage data, where some countries report data quality challenges in areas of instability or frequent population migration. Even though risk factors such as age, sex, compliance with MDA, and use of bednets have been shown to influence infection in individuals [40,48–50], we could not include factors from the human host domain in our analysis, as data sets were aggregated at site level and did not include individual information. In addition, vector control data were not universally available across the 13 countries and thus were not included in the analysis, despite studies showing that vector control has an impact on reducing LF prevalence [41,48,51–53].

Fourteen districts were excluded from the analysis because we were not able to obtain complete data for baseline prevalence, MDA coverage, or geographic boundaries. One of these districts had failed pre-TAS. It is likely these exclusions had minimal impact on the conclusions, as they represented a small number of districts and were similar to other included districts in terms of key variables. Unreported data could have occurred if a country conducted a pre-TAS that failed and then chose not to report it or reported it as a mid-term survey instead. Anecdotally, we know this has occurred occasionally, but we do not believe the practice to be widespread. Another limitation in the analysis is a potential misassignment of key variable values to a district due to changes in the district over time. Redistricting, changes in district size or composition, was pervasive in many countries during the study period; however, we expect the impact on the study outcome to be minimal, as the historical prevalence and MDA data from the “mother” districts are usually flowed down to these new “daughter” districts. However, it is possible that the split created an area of higher prevalence or lower MDA coverage than would have been found on average in the overall larger original “mother” district. Finally, the aggregation or averaging of results to the district level may mask heterogeneity within districts. Though this impact could be substantial in districts with considerable heterogeneity, the use of median values and binomial variables mitigated the likelihood of skewing the data to extreme outliners in a district.

As this analysis used data across a variety of countries and epidemiological situations, the results are likely relevant for other districts in the countries examined and in countries with similar epidemiological backgrounds. In general, as more data become available at site level through the increased use of electronic data collection tools, further analysis of geospatial variables and associations will be possible. For example, with the availability of GPS coordinates, it may become possible to analyze outcomes by site and to link the geospatial environmental domain variables at a smaller scale. Future analyses also might seek to include information from coverage surveys or qualitative research studies on vector control interventions such as bed net usage, MDA compliance, population movement, and sub-populations that might be missed during MDA. Future pre-TAS using electronic data collection could include sex and age of individuals included in the survey.

This paper provides evidence from analysis of 554 districts and 13 countries on the factors associated with pre-TAS results. Baseline prevalence, elevation, vector, population density, EVI, rainfall, and number of MDA rounds were all significant in either bivariate or multivariate analyses. This information along with knowledge of local context can help countries more effectively plan pre-TAS and forecast program activities, such as the potential need for more than five rounds of MDA in areas with high baseline and/or low elevation.
